# Scalp dysaesthesia and lichen simplex chronicus: diagnostic and therapeutic update with literature review

**DOI:** 10.1111/ced.14808

**Published:** 2021-08-30

**Authors:** M. Starace, M. Iorizzo, V. D. Mandel, F. Bruni, C. Misciali, Z. Apalla, T. Silyuk, G. Pellacani, A. Patrizi, B. M. Piraccini, A. Alessandrini

**Affiliations:** ^1^ Dermatology Unit Department of Experimental, Diagnostic and Specialty Medicine (DIMES) Alma Mater Studiorum University of Bologna IRCCS Policlinico di Sant’Orsola Bologna Italy; ^2^ Private Dermatology Practice Lugano and Bellinzona Switzerland; ^3^ Dermatology Unit Surgical, Medical and Dental Department of Morphological Sciences related to Transplant, Oncology and Regenerative Medicine University of Modena and Reggio Emilia Modena Italy; ^4^ Dermatology Unit Department of Clinical and Experimental Medicine University of Parma Parma Italy; ^5^ Dermatology Department Hippokration Hospital of Thessaloniki Thessaloniki Greece; ^6^ Private Dermatology Practice Hair Treatment and Transplantation Center Saint Petersburg Russia; ^7^ Dermatologic Unit Department of Clinical Internal, Anesthesiological and Cardiovascular Sciences Sapienza University of Rome Rome Italy

## Abstract

Scalp dysaesthesia, considered a variant of the cutaneous dysaesthesia syndrome, is characterized by chronic sensory symptoms, including pruritus, pain, burning and stinging in a well‐defined location, without objective findings. Its aetiology is not well elucidated and treatment options are limited, thus it can be challenging and frustrating for both patient and physician. It can be associated with lichen simplex chronicus. In this paper, we review the literature on the pathogenetic factors, diagnostic methods and therapeutic options in the management of scalp dysaesthesia. Dissociation, cervical spine disease and muscle tension seem to be the most important pathogenetic factors. Trichoscopy, reflectance confocal microscopy and biopsy are all helpful for the diagnosis of the disease. Therapies include high‐potency topical or intralesional corticosteroids, capsaicin and topical anaesthetics, sedative antihistamines, tricyclic antidepressants, transcutaneous electric nerve stimulation, botulinum toxin and vitamin B12.

## Introduction

Scalp dysaesthesia (SD) is considered a variant of the cutaneous dysaesthesia syndrome, and is characterized by chronic sensory symptoms, including pruritus, pain, burning and stinging in a well‐defined location, without objective findings.^1^, ^2^ The quality of life of patients suffering from SD may be severely affected. There is a marked female predominance, which is remarkable, with a peak age incidence of 35–50 years. Because the symptoms are localized to the skin, patients often present to dermatologists, and it is therefore important that dermatologists are familiar with this condition and its underlying causes.

## Pathogenesis

In terms of pathogenesis, SD has been linked to an underlying psychopathological background or to chronic pain syndrome. Localized radicular neuropathy and abnormal nerve conduction have been shown to be related to SD. Cervical spine involvement,^3^, ^4^ in which a hyperexcitable state may interfere with pathways descending from an inhibitory centre responsible for pain and pruritus modulation, has also been shown to be associated with SD. While both pain and itch are induced by chemical messengers (histamine, tissue proteases and prostaglandins), there is evidence indicating that a unique subpopulation of unmyelinated C nerve fibres are directly activated by pruritus‐inducing stimuli.^5^ Other potential triggers of SD are stress, hairstyle, heat and seasonality. Psychological stress^6^ may directly affect the skin via psychoneuroimmunological reactions due to cutaneous reactivity and sympathetic activation coordinated by the hypothalamic–pituitary–adrenal (HPA) axis. The HPA axis plays a central role in the regulation of epidermal barrier function, cutaneous immune function and cutaneous adnexal and dermal functions, maintaining local and systemic homeostasis.^6^ Psychological stress in patients with SD may be also aggravated by disorders such as cervical spine disease (mostly a degenerative disc disease) and muscle tension due to incorrect posture, resulting in nerve compression, which is correlated with the dermatomal distribution of pruritus.^3^, ^4^ Consequently, the patient’s medical history, habits, social background and environmental factors should be thoroughly investigated to assist in correct diagnosis and appropriate management of this frustrating condition.^7^, ^8^, ^9^

### Differential diagnoses

Owing to its symptoms, SD is often misinterpreted in the early stages as seborrhoeic dermatitis or red scalp syndrome, with subsequent delayed diagnosis and inadequate treatment. As SD can be intensively pruritic, it can result in subsequent development of lichen simplex chronicus (LSC), which appears as single or multiple, clearly demarcated, thickened and hyperpigmented plaques, with scaling and alopecia due to hair breakage.^10^, ^11^ However, LSC mostly involves the forearms, scrotum and shins, whereas scalp localization is rare.^12^ Clinical examination usually reveals single or multiple, irregular, polycyclic or oval‐shaped, well‐demarcated, thickened and hyperpigmented plaques. Presence of scales and alopecia due to hair breakage are not uncommon. LSC is the result of chronic and repetitive scratching. Scarring is rare, but can be observed in elderly patients.

**Figure 1:**
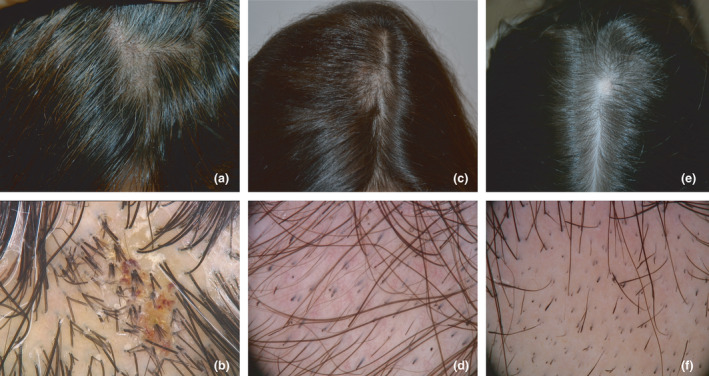
(a) Clinical and (b) trichoscopic (original magnification × 40) features of lichen simplex chronicus, showing short hair shafts with split ends and broom hair. (c,e) Clinical and (d,f) trichoscopic (original magnification × 20) features of (c,d) trichotillomania, showing flame hair, tulip hair and broken hair of different lengths; and (e,f) alopecia areata, showing black dots, dystrophic hair and exclamation‐mark hair.

LSC may be misdiagnosed as trichotillomania, tinea capitis or alopecia areata, but trichoscopy is a useful aid to the differential diagnosis. The typical trichoscopic signs of LSC and their correlation with the corresponding pathological features were described in 2017 by Rakowska *et al*.,^13^ and include short hair shafts with split ends that emerge in a group of 2–4 hairs from a single follicular opening, acquiring the aspect of a broom (broom hair), and short hair shafts of the same length with broken tips due to trichorrhexis. In LSC, the skin is also usually thickened and hyperpigmented (postinflammatory hyperpigmentation). Trichoscopy allows discrimination between LSC and trichotillomania, in which the short broken hairs are of different lengths, and between LSC and alopecia areata, as the latter is characterized by black dots, dystrophic hair and exclamation‐mark hairs^14^ (Fig. 1). The short hairs of trichorrhexis nodosa must be differentiated from the Morse code‐like hairs typically seen in tinea capitis, in which the multiple, whitish, transverse interruptions along the shaft are due to fungal penetration.^15^

Histopathologically, the trichoscopic signs of LSC correspond to hyperkeratosis of the infundibular ostium, with hair shafts split into two parts by a layer of red blood cells, a feature known as the ‘hamburger sign’.^16^, ^17^ However, this can also be observed in trichotillomania. Typical of LCS is the formation, at the level of the infundibulum, of jagged acanthotic projections that, together with the hair canal in the middle, resemble a ‘gear wheel’ on horizontal sections.^16^, ^17^ In the uppermost sections, the epidermis shows thick layers of orthokeratosis and hyperkeratosis. The follicular architecture and the terminal to vellus hair ratio are preserved, with a normal number of terminal follicles, but with a decrease in size and number of sebaceous glands.^16^, ^17^

In a recent paper, we also described the role of reflectance confocal microscopy (RCM) in LSC to facilitate the differential diagnosis between scarring and nonscarring alopecias, biopsy site selection and treatment monitoring.^18^ In some cases RCM may be particularly informative, allowing diagnosis without the need of a biopsy.

### Management

Management of SD, with or without LSC, is extremely challenging and often frustrating for both patient and clinicians. The goal of treatment is to interrupt the vicious cycle of itching.^19^ In the scenario of a neurological trigger of any kind, a neurological consultation is advisable before starting treatment. Supportive counselling, comprising many different psychological interventions may be needed in severe cases. In combination with medical therapy, simple exercises of stretching, once or twice a day, may help to improve cervical nerve compression through the restoration of normal cervical homeostasis.^20^

High‐potency topical (with or without occlusion) or intralesional corticosteroids are usually effective, but their prolonged use as maintenance treatment is limited by their well‐known adverse effects (AEs).

Topical treatments such as capsaicin in concentrations ranging from 0.025% to 0.1% (with 0.075% reported as optimal), tacrolimus 0.1%, salicylic acid 3–5%, lidocaine and compounds including lidocaine, such as TALK (topical amitriptyline 5%, lidocaine 5% and ketamine 10%) have with slow dose escalation for better tolerance, have been used with satisfactory results.^21^, ^22^ Creams and foams are generally better tolerated than lotions. At least twice‐daily application is needed but it may take 4–6 weeks for effectiveness.

Systemic treatments include sedative antihistamines as supportive treatment, as they are not sufficient as monotherapy. Low doses of oral pregabalin^23^ (up to 300 mg/day), gabapentin (up to 3000 mg/day), mirtazapine (15 mg/day), amitriptyline (10–25 mg/day), doxepin (up to 280 mg/day)^24^ and naltrexone (3–5 mg/day) are all possible options, but AEs, especially sedation, hypotension and anticholinergic effects, may limit their use. A list of the possible treatment options is summarized in Table 1.

**Table 1:** Suggested treatments for scalp dysaesthesia and lichen simplex chronicus.

Treatment	Dosage
Topical	
Triamcinolone acetonide^a^	2.5 mg/mL once monthly
Topical clobetasol^b^	Once a day
Topical capsaicin (cream)	0.025–0.1% 3 times daily
TALK^c^	Three times daily
Topical tacrolimus	0.1% twice daily
Topical salicylic acid	3–5% twice daily
Topical gabapentin	10% 3 times daily
Systemic treatment	
Anticonvulsants and antidepressants	
Pregabalin	50–300 mg daily
Gabapentin	Up to 3000 mg daily
Mirtazapine	15 mg daily
Naltrexone	3–5 mg daily
Amitriptyline	10–25 mg daily
Doxepin	Up to 280 mg daily
Alprazolam	Start at 0.25 mg daily
Lorazepam	Start at 1 mg daily
Sertraline	Start at 50 mg daily
Antihistamines	
Bilastine	20–60 mg daily
Hydroxyzine hydrochloride	25–50 mg daily
Cetirizine	10–30 mg daily
Levocetirizine	5–15 mg daily
Loratadine	10–30 mg daily
Fexofenadine	60–360 mg daily

^a^ Intralesional injections;

^b^ cream with or without occlusion;

^c^ topical amitriptyline 5%, lidocaine 5% and ketamine 10% in Lipobase^®^.

In addition, transcutaneous electric nerve stimulation^25^ and botulinum toxin^26^ have been reported to effectively reduce itch, but they are generally reserved for severe or recalcitrant cases and require the contribution of a neurologist. Supplementation with vitamin B12, if serum levels are < 550 pg/mL, may induce improvement of the SD associated with telogen effluvium, reducing the shedding.^27^, ^28^, ^29^

## Conclusion

Both SD and LSC are challenging conditions in terms of diagnosis and management, mostly due to their vague aetiopathogenesis and the lack of well‐established treatments. Psychogenic and neurogenic factors play a crucial role and should be thoroughly investigated. Muscular tension in the neck and shoulders (trapezius and sternocleidomastoid muscles) should always be considered as a potential trigger factor for scalp itch/pain and should be treated accordingly in order to improve the overall outcome. In terms of treatment, the potent topical or intralesional steroids are the first‐line choice. Other treatments have less evidence to recommend them, but lack of evidence does not by definition mean lack of efficacy, and so these may have value for patients unresponsive to steroid treatment.

## Acknowledgement

Open Access Funding provided by Universita degli Studi di Bologna within the CRUI‐CARE Agreement. [Correction added on 18 May 2022 , after first online publication: CRUI funding statement has been added.]

**Learning points**

- SD is a condition of chronic pain, burning/stinging sensation and/or pruritus of the scalp, in the absence of related skin or systemic disease.
- A neurological and psychological aetiopathogenesis must be investigated, thus referral to a specialist is essential.
- SD may result in LSC, which appears as single or multiple, well‐demarcated, thickened and hyperpigmented plaques, with scaling and alopecia due to hair breakage.
- Trichoscopy is fundamental to the diagnosis of LSC, and histopathological confirmation may also be needed.
- Therapy of SD is based on high‐potency topical or intralesional corticosteroids, capsaicin and topical anaesthetics, sedative antihistamines, tricyclic antidepressants, transcutaneous electric nerve stimulation, botulinum toxin and vitamin B12.

## CPD questions

### Learning objective

To gain up‐to‐date knowledge on the features, pathogenesis and diagnosis of scalp dysaesthesia and lichen simplex chronicus.

### Question 1

What are the most common symptoms of scalp dysaesthesia (SD)?

(a) Pruritus and erythema.
(b) Burning and pain.
(c) Stinging and pustules.
(d) Blisters and erythema.
(e) Pruritus, burning and stinging.

### Question 2

In which of the following groups is scalp dysaesthesia (SD) most frequently seen?

(a) Female patients aged 35–50 years.
(b) Male patients aged 35–50 years.
(c) Female patients aged 50–70 years.
(d) Male patients aged 20–30 years.
(e) Male patients aged 10–19 years.

### Question 3

Which of the following is/are the only factor(s) involved in the pathogenesis of scalp dysaesthesia (SD)?

(a) Inflammation of the hair follicle.
(b) Metabolic syndrome.
(c) Psychological stress.
(d) Local trauma.
(e) Abnormal nerve conduction, cervical spine involvement and psychological stress.

### Question 4

Which of the following characterizes the trichoscopy feature(s) of lichen simplex chronicus (LSC)?

(a) Patchy hair loss with exclamation‐mark hair.
(b) Scarring alopecia with red dots.
(c) Hair loss with demarcated thickened and hyperpigmented plaques, scaling and alopecia due to hair breakage.
(d) Comma hair.
(e) Corkscrew hair.

### Question 5

Which of the following should be considered in the differential diagnosis of lichen simplex chronicus (LSC)?

(a) Telogen effluvium.
(b) Androgenetic alopecia.
(c) Dissecting cellulitis.
(d) Frontal fibrosing alopecia.
(e) Trichotillomania and alopecia areata.

## Instructions for answering questions

This learning activity is freely available online at http://www.wileyhealthlearning.com/ced

Users are encouraged to

- Read the article in print or online, paying particular attention to the learning points and any author conflict of interest disclosures.
- Reflect on the article.
- Register or login online at http://www.wileyhealthlearning.com/ced and answer the CPD questions.
- Complete the required evaluation component of the activity.

Once the test is passed, you will receive a certificate and the learning activity can be added to your RCP CPD diary as a self‐certified entry.

This activity will be available for CPD credit for 2 years following its publication date. At that time, it will be reviewed and potentially updated and extended for an additional period.
